# Leg ulcer nursing care in the community: a prospective cohort study of the symptom of pain

**DOI:** 10.1186/1472-6955-12-3

**Published:** 2013-02-06

**Authors:** Elizabeth G VanDenKerkhof, Wilma M Hopman, Meg E Carley, Janet L Kuhnke, Margaret B Harrison

**Affiliations:** 1 School of Nursing, Queen’s University, Kingston, ON, Canada; 2 Department of Anesthesiology, Queen’s University, Kingston, ON, Canada; 3 Clinical Research Centre, Kingston General Hospital, Kingston, OntarioCanada; 4 Department of Community Health and Epidemiology, Queen’s University, Kingston, OntarioCanada; 5 St. Lawrence College/Laurentian University, Cornwall, OntarioCanada

**Keywords:** Pain, Health-related quality of life, Chronic conditions, Leg ulcers, Community care nursing, Longitudinal study, Canada

## Abstract

**Background:**

Chronic wounds are managed almost entirely by community nurses. Almost all individuals with leg ulcers report acute pain usually related to dressing change. Little is known about pain after healing. The purpose of this study was to explore the course of pain from baseline to time of healing of leg ulcers (venous or mixed etiology). In order to understand this phenomenon and develop implications for nursing practice, objectives included: 1) Measure incidence and prevalence of pain at baseline and healing; 2) Describe characteristics associated with leg ulcer pain at baseline; 3) Identify predictors of leg ulcer pain at healing.

**Methods:**

Data were from one randomized controlled trial (2004-2008) of 424 individuals with leg ulcers in the community receiving evidence-informed nursing management. The primary outcome was pain at the time of healing. Predictive factors included demographic, circumstance of living, clinical and ulcer characteristics. Multivariable logistic regression identified the subset of predictors of pain at healing. Odds ratios (OR) and 95% confidence intervals (CI) are reported.

**Results:**

Eighty-two percent of participants reported pain at baseline and 32% at healing. Five percent with no pain at baseline reported pain at healing. Thirty-seven percent reported moderate to severe pain at baseline and 11% at healing. Twenty percent of all those who healed reported pain interfered with work moderately to extremely at time of healing. Being female (OR=1.64, 95% CI 1.00, 2.68, p=0.05), use of short-stretch vs. four-layer bandages (OR=1.73, 95% CI 1.06, 2.82, p=0.03), lower SF-12 PCS (OR=0.97, 95% CI 0.94, 0.99, p=0.02) and MCS (OR=0.98, 95% CI 0.95-1.00, p=0.04) scores, use of non-steroidal anti-inflammatory drugs (OR=2.28, 95% CI 1.06, 4.88, p=0.03), and tender pain (OR=2.17, 95% CI 1.29, 3.66, p=<0.01) were associated with pain at time of healing.

**Conclusions:**

Pain is an issue on admission for chronic wounds and at healing, yet 58% with moderate to severe pain on admission were not taking pain medication(s). Future studies should examine the role of pain at healing and at subsequent ulcer recurrence. Mobility and other factors that may contribute to pain at time of healing should also be assessed. Community nurses are encouraged to consider pain when planning care on admission and also after wound healing, when most patients are discharged from care.

**Trial registration:**

ClinicalTrials.gov, NCT00202267

## Background

Pain is one of the five most common reasons for a patient to see a physician. Recent Canadian studies indicate a prevalence of chronic pain of 18%-35%, 80% of which is moderate or severe [1-4]. In the United States, it is estimated that chronic pain costs approximately $560-$635 billion/year (~$60 billion/year in Canada) in direct medical treatment costs and lost productivity [5]. In Canada, pain treatment facility wait-list times often exceed 6-8 months [6], with most patients suffering from severe pain and depression [7]. Health-related costs for individual wait-listed patients often exceed $20,000 annually [8]. These numbers reaffirm the urgent need to further advance efforts towards the prevention and treatment of persistent pain [9].

Pain is defined by the International Association for the Study of Pain as “An unpleasant sensory and emotional experience associated with actual or potential tissue damage, or described in terms of such damage” (http://www.iasp-pain.org last accessed Feb 8, 2013). Chronic pain is generally defined as pain lasting beyond tissue healing with duration of at least 3 months. There is growing evidence that pain persists beyond normal healing time after various surgical procedures, and that certain biopsychosocial factors increase the risk of chronic postsurgical pain [10-14]. Few studies have explored potential risk factors and the prevalence and nature of pain during and after healing of chronic wounds, such as leg ulcers. Reports on the prevalence of pain with leg ulcers range from 28% to 93% [15-19], however most studies examine acute pain related to dressing changes. Examining pain reported in the absence of manipulation due to dressing change or after wound healing may be indicative of a chronic pain condition. Pain was a theme reported by 2 of 10 participants in a qualitative study of persons living with healed venous leg ulcers [20]. In a prospective study examining pain and wound healing (venous, mixed venous/arterial, lymphedema-related, and leg and foot ulcers), 52 participants with healed wounds reported an average pain intensity score of 1.67 (out of 10) after healing [21].

Managing pain with leg ulcers is primarily the role of community care nurses providing leg ulcer care in the home setting [22-25]. Up to 50% of community care-nursing time is spent caring for individuals with ulcers [23,26-28], and while there is some evidence of acute pain during dressing changes, there is a paucity of information on the trajectory of pain over the course of wound healing. The purpose of this study was to describe pain at entry to treatment and at time of healing in a large cohort of community dwelling adults treated for leg ulcers (venous or mixed etiology). The specific objectives were to: 1) Measure the incidence and prevalence of pain on admission to community care (baseline) and at time of healing; 2) Describe the characteristics associated with leg ulcer pain at baseline; 3) Identify baseline predictors of leg ulcer pain at time of healing.

## Methods

Individuals with leg ulcers enrolled in a randomized controlled trial between 2004 and 2008 formed the study sample [29]. The primary outcome of the original study was time to ulcer healing (fully epithelialized, no scab, no drainage), with pain and health-related quality of life (HRQL) as secondary outcomes. Healing measures were collected as close to the first observation of healing as possible, typically the same day or the following visit. Baseline and healing assessment data from the trial formed the dataset for the current study. Ethics approval for the trial was received from Queen's University Research Ethics Board, Kingston, Ontario, Canada (REB# NURS-140-03).

As a pragmatic nursing trial several procedures are noteworthy. All participants received a comprehensive evidence-based assessment by their specially trained attending registered nurses in the community care settings located in multiple regions across Canada, including urban centers as well as remote/rural areas. Inclusion criteria for the study were: adult (≥18 years), English-speaking or with access to translation, able to provide written informed consent, clinical presentation of venous insufficiency with an ankle brachial pressure index (ABPI) ≥0.8, and a leg ulcer with minimum duration of one week that measured at least 0.7 cm in any one dimension. Ulcers smaller than 0.7 cm were excluded. Very small ulcers may be difficult to distinguish from skin erosions due to varicose eczema, and are likely to heal rapidly and may not require treatment with compression. Given the reality in community nursing care with this population, an ulcer could be either a first occurrence or recurrent ulcer. Those with medication-controlled diabetes mellitus were excluded. Full trial protocol details are described elsewhere [29].

For the current study the primary outcome was the prevalence of pain at time of healing. Prevalence was defined as the percent of participants with pain at time of healing. The secondary outcome was the incidence of pain at time of healing. Incidence was defined as the percent of participants with pain at time of healing of those who were free of pain at treatment initiation. Pain specific to the leg ulcer was assessed using the short form McGill Pain Questionnaire (SF-MPQ) [30-32]. The McGill Pain Questionnaire consists of 15 pain descriptors (11 sensory and 4 affective), a visual analogue scale (VAS), and the Present Pain Intensity (PPI). Each pain descriptor can be valued either as “0=none”, “1=mild”, “2=moderate”, or “3=severe”. For the purposes of the analysis values 1 through 3 were recoded as “1-3=present” versus “0=not present”. From the descriptors, sensory, affective, and total pain index scores were generated and standardized out of 100. The VAS is a 100mm scale anchored with “0=no pain” and “10=worst possible pain”. Due to the highly skewed nature of the VAS score, pain was classified in two ways: i) present (VAS>0/10) or absent (VAS = 0); and ii) none/mild (VAS≤3/10) or moderate/severe (VAS>3/10). The PPI is comprised of six number-word combinations ranging from “0=no pain” to “5=excruciating pain”. The SF-MPQ is designed to assess the multidimensional nature of the pain experience and has been demonstrated to be a reliable, valid, and consistent measurement tool [31]. Importantly, it has been used in studies of individuals with leg ulcers and other chronic wounds and found to be a sound approach for clinically assessing the quality of pain with this population [15].

Predictive factors captured on admission for nursing care (baseline) included demographic and clinical characteristics, and HRQL. Demographic characteristics included age, sex, and circumstance(s) of living. Clinical characteristics included size and duration of ulcer at baseline, time to healing of current ulcer, and mobility and medications for pain control. Baseline characteristics were collected during the comprehensive baseline assessment using interviews and the Leg Ulcer Assessment Tool (LUAT) [33]. HRQL was measured at baseline using the Medical Outcomes Survey Short Form-12 (SF-12) [34]. The SF-12 produces two scores, the Physical Component Summary (PCS) and the Mental Component Summary (MCS). The PCS and MCS are standardized to a mean of 50, with a score above 50 representing better than average and below 50 indicating poorer than average function [34]. A two to three point difference in summary scores is considered clinically meaningful [34]. Individual missing items for the SF-12® were imputed using assignment of mean score (AMS) [35,36].

Chi-square tests were used to assess the association between categorical variables and the 2-level VAS pain measures at baseline and at time of healing, while independent t-tests were used for continuous variables. Variables with a non-normal distribution were analyzed with appropriate non-parametric procedures, Mann–Whitney *U* test for unpaired data and Wilcoxon signed ranks test for paired data. Multivariable logistic regression was then used to identify the subset of significant predictors of pain at healing. The primary outcome for the predictive model was defined as pain present at time of healing (VAS>0/10). All regression procedures used simultaneous entry. Variables were eliminated one at a time in successive regressions if p≥0.10, and retained if p<0.10 so as not to miss clinically important trends. Odds ratios (OR) and 95% confidence intervals (CI) are reported for the final multivariable model. Analyses were conducted using IBM© SPSS© (version 20 for Windows).

## Results

**Figure 1:**
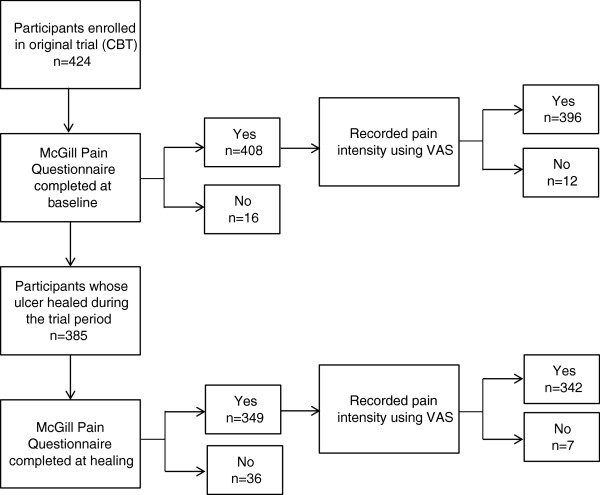
Recruitment and data collection.

Of the 424 participants enrolled in the nursing care trial, 396 recorded their pain intensity using the VAS. Of 385 who healed during the trial period, 342 recorded their pain intensity using the VAS (Figure 1). The majority (91%) of participants were English-speaking, female (55%), not living alone (64%) and fully mobile (79%) (Table 1).

**Table 1:** The association between baseline characteristics and pain at baseline (moderate to severe) and time of healing (any pain)

			**Baseline**		**Healing**	
**Characteristics**^1^		**Total**	**VAS** ≤ **3**	**VAS > 3**	**VAS=0**	**VAS > 0**
		**(n=396)**	**(n=250)**	**(n=146)**	**(n=231)**	**(n=111)**
Sex	Female	216 (54.5)	134 (62.0)	82 (38.0)	115 (62.8)	68 (37.2)
	Male	180 (45.5)	116 (64.4)	64 (35.6)	116 (73.0)	43 (27.0)
Living situation	Alone	144 (36.4)	**102 (70.8)**	**42 (29.2)**	85 (66.4)	43 (33.6)
	With others	252 (63.6)	**148 (58.7)**	**104 (41.3)**	146 (68.2)	68 (31.8)
Fully mobile	Yes	312 (78.8)	197 (63.1)	115 (36.9)	185 (68.8)	84 (31.2)
	No	84 (21.2)	53 (63.1)	31 (36.9)	46 (63.0)	27 (37.0)
Non-venous health history	Yes	240 (60.6)	143 (59.6)	97 (40.4)	142 (70.0)	61 (30.0)
	No	156 (39.4)	107 (68.6)	49 (31.4)	89 (64.0)	50 (36.0)
Leg ulcer pain on admission	Yes	344 (86.9)	**198 (57.6)**	**146 (42.4)**	**187 (64.7)**	**102 (35.3)**
	No	52 (13.1)	**52 (100.0)**	**0 (0.0)**	**36 (94.7)**	**2 (5.3)**
Pain at rest/night pain	Yes	26 (6.6)	**11 (42.3)**	**15 (57.7)**	15 (62.5)	9 (37.5)
	No	370 (93.4)	**239 (64.6)**	**131 (35.4)**	216 (67.9)	102 (32.1)
Medications for leg ulcer pain	Yes	103 (26.0)	**41 (39.8)**	**62 (60.2)**	61 (64.2)	34 (35.8)
	No	293 (74.0)	**209 (71.3)**	**84 (28.7)**	170 (68.8)	77 (31.2)
Intervention	4LB	197 (49.7)	128 (65.0)	69 (35.0)	121 (72.5)	46 (27.5)
	SSB	199 (50.3)	122 (61.3)	77 (38.7)	110 (62.9)	65 (37.1)
# Co-morbidities	None	69 (17.4)	49 (71.0)	20 (29.0)	42 (70.0)	18 (30.0)
	1 - 2	249 (62.9)	155 (62.2)	94 (37.8)	145 (68.1)	68 (31.9)
	≥ 3	78 (19.7)	46 (59.0)	32 (41.0)	44 (63.8)	25 (36.2)
Previous leg ulcers	Yes	178 (44.9)	**99 (55.6)**	**79 (44.4)**	100 (69.0)	45 (31.0)
	No	218 (55.1)	**151 (69.3)**	**67 (30.7)**	131 (66.5)	66 (33.5)
Previous compression	Yes	237 (59.8)	**134 (56.5)**	**103 (43.5)**	133 (65.8)	69 (34.2)
	No	159 (40.2)	**116 (73.0)**	**43 (27.0)**	98 (70.5)	41 (29.5)
Reference ulcer leg	Right	197 (49.7)	121 (61.4)	76 (38.6)	120 (66.7)	60 (33.3)
	Left	199 (50.3)	129 (64.8)	70 (35.2)	111 (68.5)	51 (31.5)
Edema on affected leg	Yes	334 (85.2)	214 (64.1)	120 (35.9)	192 (66.0)	99 (34.0)
	No	58 (14.8)	36 (62.1)	22 (37.9)	36 (75.0)	12 (25.0)
Full flexion on affected leg	Yes	315 (81.0)	203 (64.4)	112 (35.6)	185 (68.0)	87 (32.0)
	No	74 (19.0)	42 (56.8)	32 (43.2)	42 (65.6)	22 (34.4)
Age (years)*		65.0 (16.7)	65.4 (16.7)	64.4 (16.8)	65.8 (16.7)	63.9 (16.7)
Duration of current ulcer (weeks)	≤ 12	206 (52.0)	125 (60.7)	81 (39.3)	129 (69.4)	57 (30.6)
	> 12	190 (48.0)	125 (65.8)	65 (34.2)	102 (65.4)	54 (34.6)
Time to healing	days^†^	62 [36/146]	69 [36/178]	61 [35/100]	63 [36/136]	62 [37/172]
Area (cm^2^) – Tracing	≤ 2.5 cm	162 (41.9)	**91 (56.2)**	**71 (43.8)**	95 (68.8)	43 (31.2)
	> 2.5 to ≤ 10 cm	145 (37.5)	**101 (69.7)**	**44 (30.3)**	87 (67.4)	42 (32.6)
	> 10 cm	80 (20.7)	54 (67.5)	**26 (32.5)**	45 (66.2)	23 (33.8)
ABPI on affected leg*		1.05 (0.15)	1.04 (0.15)	1.06 (0.15)	1.06 (0.15)	1.04 (0.14)
McGill Pain Indices^†^	Sensory	15.2 [6.1/27.3]	**9.1 [3.0/18.2]**	**27.3 [18.2/42.4]**	**12.1 [3.0/24.2]**	**18.2 [9.1/30.3]**
	Affective	0.0 [0.0/8.3]	**0.0 [0.0/0.0]**	**8.3 [0.0/25.0]**	**0.0 [0.0/8.3]**	**0.0 [0.0/8.3]**
	Total	11.1 [4.4/22.2]	**6.7 [2.2/13.3]**	**22.2 [15.6/35.6]**	**8.9 [2.2/20.0]**	**13.3 [6.7/26.7]**
	PPI-VAS	2.0 [0.7/4.2]	**1.0 [0.20/1.8]**	**5.0 [3.7/6.4]**	**1.8 [0.50/3.6]**	**2.3 [1.1/4.6]**
SF-12 Component Summary Scores*	Physical	39.0 (9.9)	39.6 (9.7)	38.1 (10.0)	**40.3 (9.5)**	**37.3 (9.7)**
	Mental	51.5 (9.9)	**53.3 (9.4)**	**48.3 (9.9)**	51.9 (10.0)	49.8 (10.2)
Prescribed non-narcotics	Yes	86 (21.7)	**38 (44.2)**	**48 (55.8)**	45 (58.4)	32 (41.6)
	No	310 (78.3)	**212 (68.4)**	**98 (31.6)**	186 (70.2)	79 (29.8)
Non-narcotics for leg ulcer pain	Yes	55 (64.0)	21 (38.2)	34 (61.8)	29 (58.0)	21 (42.0)
	No	31 (36.0)	17 (54.8)	14 (45.2)	16 (59.3)	11 (40.7)
Prescribed NSAIDS	Yes	44 (11.1)	25 (56.8)	19 (43.2)	18 (52.9)	16 (47.1)
	No	352 (88.9)	225 (63.9)	127 (36.1)	213 (69.2)	95 (30.8)
NSAIDS for leg ulcer pain	Yes	11 (25.6)	4 (36.4)	7 (63.6)	4 (40.0)	6 (60.0)
	No	32 (74.4)	20 (62.5)	12 (37.5)	14 (60.9)	9 (39.1)
Prescribed Opiods	Yes	31 (7.8)	**8 (25.8)**	**23 (74.2)**	17 (63.0)	10 (37.0)
	No	365 (92.2)	**242 (66.3)**	**123 (33.7)**	214 (67.9)	101 (32.1)
Opiods for leg ulcer pain	Yes	16 (53.3)	**1 (6.3)**	**15 (93.8)**	8 (57.1)	6 (42.9)
	No	14 (46.7)	**7 (50.0)**	**7 (50.0)**	8 (66.7)	4 (33.3)

^1^Bolded values indicate p-value < 0.05 when comparing pain ≤ or > 3 at Baseline or when comparing pain = or > 0 at Healing; values in parentheses are percentages unless indicated otherwise; frequency values may not always total 100% due to missing data. *values are mean (s.d.); ^†^values are median [percentiles];. 4LB=four-layer bandage; SSB=Short-stretch bandage; ABPI=Ankle Brachial Pressure Index; PPI=Present Pain Intensity; VAS=Visual Analogue Scale.

### Pain at baseline

**Figure 2:**
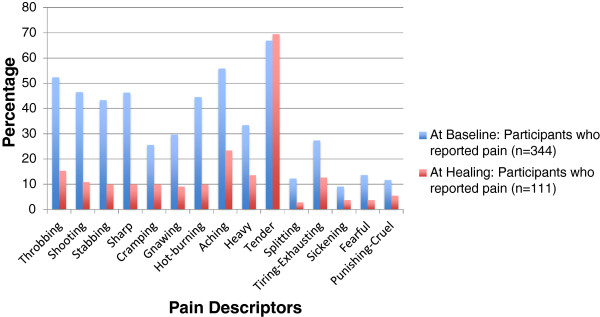
Baseline pain descriptor frequencies for the participants who reported pain at baseline and for the participants who reported pain at healing.

The prevalence of leg ulcer pain on admission for nursing care was 87%. Thirty-seven percent (146/396) reported baseline pain to be of moderate to severe intensity (VAS>3/10) (Table 1). The median sensory and affective pain indices were 15.2/100 (25^th^, 75^th^ percentile 6.1, 27.3) and 0/100 (25^th^, 75^th^ percentile 0, 8.3), respectively (Table 2). The most commonly used pain quality descriptors reported by participants with pain at baseline (VAS>0) were tender (67%), aching (56%), throbbing (52%), shooting (47%), sharp (46%), hot-burning (44%) and stabbing (43%) (Figure 2). Seventy percent (293/344) of individuals with any leg ulcer pain (VAS>0/10) and 58% (84/146) with moderate to severe (>3/10) pain at baseline were not taking medication for leg ulcer pain.

**Table 2:** Pain characteristics at baseline and time of healing

**Characteristics**^2^		**Baseline (All)**	**Healing (All)**	**Healing (Pain group)**
		**(n=408)**	**(n=349)**	**(n=111)**
McGill Pain Indices^†^	Sensory	15.2 [6.1/27.3]	0.0 [0.0/3.0]	3.0 [3.0/9.1]
	Affective	0.0 [0.0/8.3]	0.0 [0.0/0.0]	0.0 [0.0/0.0]
	Total	11.1 [4.4/22.2]	0.0 [0.0/2.2]	2.2 [2.2/6.7]
PPI-VAS^†^		2.0 [0.70/4.2]	0.0 [0.0/0.3]	0.50 [0.30/1.3]
PPI-VAS	≤3	250 (63.1)	330 (96.5)	99 (89.2)
	>3	146 (36.9)	12 (3.5)	12 (10.8)
Pain interference with normal work	Not at all	87 (21.4)	151 (43.4)	25 (22.5)
	A little bit	105 (25.9)	127 (36.5)	53 (47.7)
	Moderately	123 (30.3)	36 (10.3)	14 (12.6)
	Quite a bit	79 (19.5)	30 (8.6)	15 (13.5)
	Extremely	12 (3.0)	4 (1.1)	4 (3.6)

^2^Values in parentheses are percentages unless indicated otherwise; frequency values may not always total 100% due to missing data; †values are median [percentiles]. PPI=Present Pain Intensity; VAS=Visual Analogue Scale.

Living with others, taking medication for leg ulcer pain, a history of leg ulcers, prior compression bandaging, shorter duration of reference leg ulcer, and lower MCS scores were associated with moderate to severe pain at baseline in bivariate analysis (Table 1). Rest and night pain were also more likely to be present in individuals with moderate to severe pain at baseline. Participants who were prescribed non-narcotic analgesia and/or opioids were more likely to report moderate to severe pain at baseline (Table 1).

### Pain at healing

The prevalence of leg ulcer pain at time of healing was 32% and 3.5% reported moderate to severe pain. The incidence of new leg ulcer pain at time of healing was 5.3%. The median pain sensory and affective scores for the 111 respondents reporting pain at time of healing was 3/100 (25^th^, 75^th^ percentile 3.0, 9.1) and 0/100 (25^th^, 75^th^ percentile 0, 0), respectively (Table 2). Twenty percent of all those who healed reported pain interfered with work moderately to extremely. The most commonly reported pain quality descriptors were tender (69%), aching (23%), throbbing (15%), heavy (14%), tiring-exhausting (13%), shooting (11%), and sharp (10%) (Figure 2).

Baseline pain (VAS>0) was the strongest predictor of pain on healing, however this resulted in an imprecise estimate, i.e., a wide confidence interval (OR=7.61; 95% CI 1.78, 32.53) because 87% of participants had pain at baseline. Therefore, we classified baseline pain into none/mild vs. moderate/severe. In bivariable analysis, moderate to severe baseline pain increased the risk of pain on healing (OR=1.69, 95% CI 1.05, 2.73), however baseline pain was no longer significant after controlling for other factors in multivariable analysis. It remained non-significant, even after removing use of non-steroidal anti-inflammatory analgesia at baseline, from the model (OR=1.41, 95% CI 0.86, 2.33). The final multivariable model included being female (OR=1.64, 95% CI 1.00, 2.68, p=0.05), use of short-stretch bandaging (OR=1.73, 95% CI 1.06, 2.82, p=0.03), lower SF-12 PCS (OR=0.97, 95% CI 0.94, 0.99, p=0.02) and MCS (OR=0.98, 95% CI 0.95-1.00, p=0.04) scores, use of non-steroidal anti-inflammatory drugs (OR=2.28, 95% CI 1.06, 4.88, p=0.03), and pain quality described as tender (OR=2.17, 95% CI 1.29, 3.66, p=<.01) (Table 3).

**Table 3:** **Baseline predictors of pain (VAS > 0) at healing**^**3**^

**Baseline variable**		**Unadjusted**		**Adjusted**	
		**Odds ratio (C.I)**	**Sig.**	**Odds ratio (C.I)**	**Sig.**
Sex	Male	1.00		1.00	
	Female	1.60 (1.01-2.53)	0.05	1.64 (1.00-2.68)	0.05
Intervention	4LB	1.00		1.00	
	SSB	1.55 (0.98-2.46)	0.06	1.73 (1.06-2.82)	0.03
SF-12 scores					
Physical component		0.97 (0.94-0.99)	0.01	0.97 (0.94-0.99)	0.02
Mental component		0.98 (0.96-1.00)	0.08	0.98 (0.95-1.00)	0.04
Prescribed NSAIDS	No	1.00		1.00	
	Yes	1.99 (0.97-4.08)	0.06	2.28 (1.06-4.88)	0.03
Pain descriptors					
Tender	None	1.00		1.00	
	Mild to severe	2.16 (1.31-3.56)	<0.01	2.17 (1.29-3.66) -4.2)	<0.01

^3^ Variables with p-value ≤0.20 from Table 1 were included in the bivariate analyses. 342 participants were included in the bivariate analysis with the exception of ABPI (n=341); PPI-VAS (n=327) Physical and Mental Component Scores (n=336); Pain descriptors (n=336). A manual backwards stepwise method was used for the multivariate analysis. Only variables with p<0.10 were included in the final model (n=336). Variables entered at Step 1 that were excluded from the final model were: ABPI, VAS score <= or >3, Prescribed Non-narcotics, Pain Descriptors Shooting, Stabbing, Sharp, Hot-Burning, Aching, Heavy, Splitting, Tiring-Exhausting, and Fearful.

## Discussion

This is the first large, quantitative, prospective study to report the trajectory of leg ulcer pain from admission to time of wound healing, when most individuals are discharged from community nursing care service. The results reveal important implications for community nursing practice and care planning.

Pain is clearly an issue for this population both at the time of admission for care and at time of healing and discharge from service. Almost all participants reported pain (87% VAS>0/10; 37% VAS>3/10) at the initiation of leg ulcer treatment and approximately one third reported pain (32% VAS>0/10; 3.5% VAS>3/10) once an ulcer was healed. Approximately 10% of the sample reported pain descriptors (e.g., burning, stabbing, shooting) that are consistent with pain of predominantly neuropathic origin. Baseline predictors of pain at healing were being female, use of short-stretch bandage to treat leg ulcers, pain quality described as tender, and lower SF-12 PCS and MCS scores. Contrary to our expectations, baseline pain was not an independent predictor of pain at time of healing, however use of non–steroidal anti-inflammatory drugs at baseline more than doubled the risk of pain at time of healing. A possible explanation may be that the taking of NSAIDs leads to reduced pain at baseline. Yet the group taking NSAIDS at baseline likely represents those with the most severe pain, and that group, conceivably could have an increased risk of ongoing pain at time of healing. The finding regarding baseline analgesia consumption being a risk factor for chronic pain is consistent with the literature on chronic postsurgical pain, where preoperative pain and opioid use are predictors of persistent pain after surgery [13]. Additional leg ulcer characteristics nurses might expect to predict pain at healing, such as duration and size of ulcer, were also not independent predictors of pain after healing. Finally, we found no other study reporting differences in pain between the two bandaging technolo-gies, therefore the finding that short-stretch bandaging increased the risk of pain at the time of healing, requires further study.

These findings are consistent with the findings in two earlier and smaller studies reporting on pain *after* healing [20,21]. Noteworthy is that the prevalence of pain *at time* of wound healing was not reported in either study. Woo (2009) reported a mean pain score of 1.67/10 in patients with healed ulcers, which is higher than the mean pain score of 0.37 in our study; This higher pain intensity rating reported by Woo may be explained by the inclusion of individuals with foot and/or leg ulcers [21]. The former is known to be more painful than the latter [16]. This ongoing presence of pain after healing supports the notion that a chronic pain condition may develop; possibly neuropathic in nature, due to potential nerve injury, but also potentially due to poorly managed acute nociceptive pain. Several studies highlight the high prevalence and lack of treatment plans to deal with pain with leg ulcers [16,37,38]. We found that 58% of those with moderate to severe pain at baseline were not taking medication for leg ulcer pain.

### Strengths and limitations

Strengths of this study include the large sample size, prospective nature, consistent evidence-informed follow-up assessments by trained nurses, and large number and range of characteristics that could be examined. Study limitations include the reduced sample size for the follow up analysis due to missing data on some of the baseline characteristics. In particular, some of the pain characteristics were not captured at baseline, possibly because pain was not the primary outcome in the original RCT. If those without pain were less likely to record pain scores, then our findings overestimate the prevalence of pain at healing by up to 3% (29% vs. 32%). Finally, it is possible that the tissue at the site of the ulcer may have remained tender due to nociceptive processes because the assessment was done at time of healing.

## Conclusions

### Practice implications and future research

Pain with leg ulcers is traditionally associated with treatment and the healing process, however our findings suggest that pain persists at time of healing, which in turn may limit or alter mobility and increase the risk of leg ulcer recurrence. The primary message for clinicians is the need to consider pain as the ‘fifth vital sign’ [39] all the way along the trajectory of healing, including at the time of healing. As accreditation and other oversight bodies include pain with vital signs assessment, it will be increasingly important from a health services and policy perspective as well as being clinically important [40]. Community care nurses should monitor and assist individuals to verbalize and manage their pain both during and after wound healing with pharmaceutical and non-pharmaceutical interventions. It will be important to consider pain issues at the time of care planning on admission as well as at follow-up. Those individuals with pain at time of healing may benefit from remaining on service or being referred to their primary care provider for ongoing follow up until pain is managed or resolves. This may reduce the likelihood of recurrence due to mobility limitations. Although pain scales (e.g. a VAS or Numeric Rating Scale (NRS) measure) are commonly used in practice, the same is not true for documentation of quality of pain. During leg ulcer care, community care nurses can enhance the monitoring of pain by asking patients to describe their pain using ‘pain descriptors’ such shooting, stabbing and burning. This may signify the presence of chronic pain conditions. This will improve the overall management and care planning when additional referrals may be needed.

With the population living with chronic wounds, future studies are needed to track the trajectory of pain from ulcer onset to beyond the time of healing. Importantly this will increase our understanding of whether pain at healing predicts the recurrence of leg ulcers, possibly due to limited mobility. Additionally, longitudinal studies are needed to establish other baseline risk factors for pain during and beyond healing. Intervention studies to better manage acute pain are required to investigate the contribution of nociceptive and/or neuropathic pain in the maintenance of pain once the ulcer is healed. In the interim, community care nurses could focus on providing effective pain management strategies during and after healing with individuals at highest risk of ongoing pain; females with low physical functioning reporting tender pain, and treatment with short stretch bandaging. The current findings provide the basis for a more comprehensive template for community care nurses to conduct their routine evidence-informed assessments of those living with chronic wounds.

## Competing interests

The authors declare that they have no competing interests.

## Authors' contributions

EGV conceptualized the analysis plan for the secondary analysis, drafted the manuscript, and was responsible for the analysis and interpretation of the results. WMH, considered an expert in the area of HRQL, contributed to the conceptualization of the secondary analysis plan, drafting of the manuscript, and analysis and interpretation of results. MBH was principal investigator of the Canadian Bandaging Trial (CBT) and responsible for the conceptualization, ethical approval, conduct and management of the CBT. She contributed to the conceptualization of the secondary analysis, interpretation of the results, and critically appraised the manuscript. MEC was responsible for data management of the CBT, contributed to the conceptualization of the secondary analysis, drafting of the manuscript, assisted with the analysis, and contributed to the interpretation of the results. JLK, a clinical expert, participated in the analysis plan, contributed to the interpretation of the results, and critically appraised the manuscript. All authors have read and approved the final manuscript.

## Pre-publication history

The pre-publication history for this paper can be accessed here:

http://www.biomedcentral.com/1472-6955/12/3/prepub
